# Growth performance and meat characteristics of the first filial Awassi-Rambouillet callipyge ram lambs

**DOI:** 10.14202/vetworld.2019.783-788

**Published:** 2019-06-11

**Authors:** Khaleel I. Z. Jawasreh, A. H. Al-Amareen, P. Y. Aad

**Affiliations:** 1 Department of Animal Production, Jordan University of Science and Technology, Irbid 22110, Jordan; 2 National Agricultural Research Center, Jordan; 3 Department of Sciences, Faculty of Natural and Applied Sciences, Notre Dame University, Lebanon

**Keywords:** Awassi sheep, callipyge, carcass composition, gene introgression, growth performance

## Abstract

**Aim:**

This study was designed to introduce the callipyge (CLPG) and 50% of Rambouillet sheep genes to improve meat quality and quantity of Awassi (AW) sheep.

**Materials and Methods:**

The CLPG mutation was introduced into the AW sheep through frozen semen of homozygous Rambouillet rams for the CLPG mutation. Four ram lambs from the first-generation Rambouillet callipyge Awassi (F1-RCA) and five from pure AW were recruited for a fattening trial conducted in individual pens using standard ration, following which ram lambs were slaughtered for carcass and meat evaluation.

**Results:**

Final body weight, dry matter intake, average daily gain, and feed conversion ratio were significantly higher in F1-RCA than AW. Hot and cold carcass weights and the other carcass cuts’ weights, except for the fat tail, were heavier in F1-RCA than AW. There was no difference in dressing percentage between the two genetic groups (p>0.05). All non-carcass components’ weights, except spleen, kidney, and testis, were higher in F1-RCA. Total lean, total bone, and intermuscular fat weight were greater in F1-RCA, but bone-to-lean ratio was lower in F1-RCA when compared with AW (p<0.01). No differences (p>0.05) were observed in all meat quality parameters for muscle longissimus with the exception of pH, redness color, and tenderness that were lower (p<0.05) in F1-RCA than AW. F1-RCA lambs had larger longissimus muscle area (30.9 vs. 16.9 cm^2^) and less leg fat depth (11.1 vs. 17.4 mm).

**Conclusion:**

The implications of this research show the potential of 50% of Rambouillet genes and the CLPG mutation to improve growth and meat characteristics in AW-Rambouillet crosses and can be used further to develop a meat-type AW with improved productivity and muscle mass.

## Introduction

Awassi (AW) is a multipurpose fat-tailed sheep breed, well adapted to harsh environmental conditions, and the predominant sheep breed in Jordan, Lebanon, and the Middle East. The AW sheep breed suffers from inferior carcass merit and poor leg muscling [1]. With consumers requiring meat with more lean and less fat without compromising juiciness and flavor [2], heavier carcasses could be advantageous for producers by providing higher profit from meat sales and for consumers by supplying more mature meat with better quality [3].

Recently, mutations affecting muscle growth and development have been identified in sheep. The exploitation of these mutations in breeding strategies has the potential to significantly improve lamb meat quality. With the AW lambs containing less dressing and more fat, genetic improvement by introducing new sires, crossbreeding, or a combination of breeding methods will allow for improving productive traits, especially growth and meat production [4]. The callipyge (CLPG) is a gene causes a muscle development mutation in sheep. It does not affect muscles at birth but leads to postnatal muscle hypertrophy of the leg and loin at 4-6 weeks postpartum [5] with no effect on diaphragm and some shoulder muscles [5-7]. The hypertrophy develops only in paternal heterozygous animals (N^mat^C^pat^, where N is the wild-type allele and C is the allele carrying the mutation), as polar overdominance [8]. The maternal heterozygote (C^mat^N^pat^) and homozygote (C^mat^C^pat^) animals show no muscling phenotypes. The CLPG locus has been mapped to the telomeric region of ovine chromosome 18, accession no. JN227865 [8,9]. CLPG lambs have superior feed efficiency [5], higher dressing percentages [6,10], and 42% more muscle mass [6,7,10] compared with normal type lambs. However, the CLPG lambs produce inherently tough meat, especially in the longissimus muscle when compared with normal lamb [6].

The Jordan University of Science and Technology has developed a long program to enhance the AW meat characteristics potential, including feed efficiency, meat growth, and tenderness by the introgression of the CLPG mutation into AW sheep. In this study, we introduced the CLPG mutation from CLPG Rambouillet into purebred AW sheep for the 1^st^ time and evaluated these animals for growth and meat characteristics.

## Materials and Methods

### Ethical approval

All procedures used in this study were approved by the Animal Care and Use Committee at Jordan University of Science and Technology (JUST), Jordan, in accordance with international standards for ethical use of animals in research.

### Animals and breeding strategies

Semen from four homozygous Rambouillet rams carrying CLPG gene was collected at the Utah University (USA), frozen, and shipped to the JUST Center for Extension and Agriculture Research. Pure AW ewes were inseminated using laparoscopic and transcervical techniques with CLPG-positive Rambouillet frozen semen during the normal mating season in Jordan to produce the first-generation Rambouillet callipyge Awassi (F1-RCA) crossbreed. The CLPG mutation was detected by Restriction Fragment Length Polymorphism-polymerase chain reaction technique and confirmed by visualizing the phenotype after 6 weeks of age.

### Fattening trial

The fattening trial of the 60±2-day-old weaned ram lambs was conducted at JUST Center for Extension and Research, following a 2-weeks adaptation period to avoid weaning stress. F1-RCA ram lambs n=4 and pure AW ram lambs n=5 from the station and of the same age and weight were housed in individual (1.5×1.75 m^2^) pens. All lambs were introduced slowly to *ad libitum* access to both water and a diet containing 16% CP and 2.78 *Mcal* metabolizable energy/kg for a period of 74 days [11]. The ingredients of the mixed ration were soybean (15%), barley (61.4%), wheat straw (21%), salt (1.5%), limestone (0.1%), and minerals and vitamins (0.1%). Feed and orts were weighed daily for the calculation of feed intake and feed efficiency. Live weights of the lambs were recorded weekly.

### Slaughtering procedures, carcass composition, and meat quality

At the end of the fattening period, after 12 h of fasting with free access to water, lambs were slaughtered (at the age of 134 days) using a standard slaughter procedure described by Yagoubi *et al*. [12]. Briefly, carcass cuts were divided into four parts (shoulder, rack, loin, and leg cuts), in addition to the fat tail [13], and all their weights were recorded. After sectioning each carcass, the rib-eye area, fat depth, tissue depth (GR), rib fat depth (J), eye muscle width (A), eye muscle depth (B), eye muscle area, fat depth (C), shoulder fat depth (S2) and leg fat depth (L3), and longissimus muscles were measured on chilled cuts [12]. Each major cut was separated into the right and left sides using an electrical saw. The right side of each cut was sealed in a plastic bag and frozen at −20°C until the dissection of the frozen right leg to determine its muscle, bone, subcutaneous fat, and intermuscular fat components as indicators to muscularity of the whole carcass. Meat quality measurements included Warner-Bratzler shear force values on cooked meat samples, water holding capacity, cooking loss, and color coordinates (L*, a*, and b*) as described by Abdullah and Qudsieh [14].

### Statistical analysis

Data were analyzed as a completely randomized design using the mixed procedure of SAS (8.1, 2000, SAS Inst. Inc., Cary, NC) where the genotype of the animals was inserted as a fixed effect and the sires as a random effect in the model. The initial weight was used as covariate for the analysis of final body weight, fasting live weight, hot and cold carcass weights, dressing percentage, and average daily gain (ADG). Data were presented as least square mean ± standard error of the means and least square difference option of the mixed procedure of SAS (8.0) was used to identify differences among means. Statistically significant differences were considered at p≤0.05.

## Results

The effects of the CLPG mutation on growth performance are presented in Table-1. No differences were observed (p>0.05) in initial body weight between the two genotypes AW and F1-RCA, whereas final body weight for F1-RCA was higher than the AW lambs (p<0.001). F1-RCA consumed significantly (p<0.05) more feed than AW lambs during the fattening period (Table-1) and showed the highest ADG as compared to AW (0.36±0.02 vs. 0.25±0.02 kg) during the fattening period. The average feed conversion ratio (FCR) recorded for F1-RCA and AW was 3.26 and 4.04 kg feed/kg gain, respectively (p<0.0187).

**Table-1:** Effect of the first-generation callipyge Rambouillet-Awassi and pure Awassi genotypes on growth performance of lambs.

Genotype				

Trait	F1-RCA	AW	p-value	*Covariate
Initial weight (kg)	27.13±2.02	24.50±1.81	0.3649	
Final body weight (kg)	52.45±1.65	43.84±1.47	0.0091	0.0008
Dry matter intake (kg)	80.10±4.50	61.39±4.03	0.0174	
ADG (kg)	0.36±0.02	0.25±0.02	0.0091	0.0312
FCR (kg feed/kg gain)	3.26±0.16	4.04±0.14	0.0187	

* Initial weight was inserted as covariate, p≤0.05 considered statistically significant. ADG=Average daily gain, FCR=Feed conversion ratio

The effect of genotype on carcass trait is displayed in Table-2. Pre-slaughter weight and hot and cold carcass weights were heavier (p<0.01), but no differences (p>0.05) were observed in dressing percentage between the F1-RCA and AW lambs. The F1-RCA carcass resulted in heavier (p<0.05) cut weights of shoulders, racks, loins, and legs, a higher average weight (p<0.05) of lungs, trachea, heart, and liver, and a lighter (p<0.01) fat tail than AW. There was no difference in mesenteric and kidney fat, spleen, kidney, and testes (p>0.05) weight between the two genotypes. Further, results obtained from dissection of the loin and leg cuts (Table-3) showed that the longissimus muscle weight was significantly higher (p<0.05) in F1-RCA than AW. Leg weight of F1-RCA lambs was heavier than AW lambs and had more total lean and bone, with higher (p<0.001) bone to lean ratio than in pure AW. Table-3 also showed higher (p<0.01) intermuscular fat in F1-RCA than AW, while there were no differences (p>0.05) between two genotypes in the subcutaneous fat.

**Table-2:** Effect of F1-RCA and pure AW on carcass and carcass cuts weights and non-carcass components in yearlings.

Genotype^1^				

Weight (kg)	F1-RCA	AW	p-value	*Covariate
Pre-slaughter	51.98±1.79	42.81±1.59	0.0098	0.0018
Hot carcass	28.75±0.84	22.99±0.75	0.0026	0.0004
Cold carcass	27.85±0.79	22.14±0.69	0.0019	0.0003
Dressing %	55.12±1.61	53.53±1.03	0.3614	0.1824

**Carcass cuts’ weight (kg)**				

Shoulders	10.97±0.86	7.49±0.76	0.0191	
Legs	10.54±0.75	6.65±0.67	0.0064	
Racks	2.99±0.24	1.80±0.21	0.0071	
Loins	3.81±0.27	2.11±0.25	0.0026	
Fat tail	0.44±0.13	2.21±0.11	<0.0001	

**Non-carcass components’ weight (kg)**				

Mesenteric fat	0.559±0.08	0.340±0.07	0.0724	
Lungs and trachea	0.745±0.07	0.534±0.06	0.0486	
Heart	0.230±0.02	0.168±0.02	0.0475	
Liver	0.895±0.07	0.630±0.07	0.0302	
Spleen	0.105±0.02	0.077±0.02	0.2381	
Kidney	0.139±0.009	0.125±0.008	0.2934	
Kidney fat	0.263±0.03	0.173±0.03	0.0741	
Testes	0.253±0.06	0.167±0.05	0.3274	

1 All data are presented as mean±SEM,

* initial weight was inserted as covariate, p≤0.05 considered statistically significant. SEM=Standard error of the mean, F1-RCA=First-generation Rambouillet callipyge Awassi, AW=Awassi

**Table-3:** Effect of genotype (F1-RCA and pure AW) on dissected loin and leg cuts, carcass measurements, and meat quality characteristics.

Genotype^1^			

Trait	F1-RCA	AW	p-value
Muscle weight (kg)			
Loin	1.930±0.14	1.000±0.12	0.0015
Longissimus	0.436±0.03	0.199±0.03	0.0007
Leg	5.175±0.38	3.322±0.34	0.0082

Fat weight (kg)			

Intermuscular	0.151±0.01	0.096±0.01	0.0147
Subcutaneous	0.496±0.08	0.562±0.07	0.5645
Total lean (kg)	3.421±0.24	1.809±0.21	0.0015
Total bone (kg)	0.818±0.03	0.629±0.03	0.0061
Bone-to-lean ratio	0.242±0.01	0.350±0.01	0.0006
Eye muscle area (cm^2^)	30.9±1.13	16.9±1.01	<.0001
Eye muscle width (mm)	83.4±1.58	62.9±1.41	<.0001
Eye muscle depth (mm)	43.3±2.58	26.9±2.30	0.0022
Leg fat depth (mm)	11.1±1.69	17.4±1.52	0.0284
Tissue depth (mm)	20.7±1.64	18.4±1.47	0.3214
Rib fat depth (mm)	7.1±1.18	9.4±1.15	0.1953
Fat depth (mm)	2.3±0.85	4.7±0.76	0.0760
Shoulder fat depth (mm)	6.7±0.90	6.4±0.80	0.7816
Shear force (kg/cm^2^)	19.0±1.11	7.25±0.99	0.0001
Cooking loss (%)	44.8±0.59	45.5±0.53	0.3868
Water holding capacity (%)	28.7±0.93	26.7±0.83	0.1533
pH	5.67±0.02	5.74±0.02	0.0247

Color evaluation			

Lightness	36.5±6.67	38.4±59.66	0.0724
Redness	2.05±0.22	3.06±0.19	0.0120
Yellowness	31.68±8.88	19.41±7.94	0.3371

1 All data are presented as mean±SEM, P≤0.05 considered statistically significant. SEM=Standard error of the mean, F1-RCA=First-generation Rambouillet callipyge Awassi, AW=Awassi

The effect of genotype on carcass measurements of the F1-RCA and AW is presented in Table-3. Eye muscle area and eye muscle width and depth of the F1-RCA were larger (p<0.05) than AW. Leg fat depth was larger (p<0.05) in AW when compared to F1-RCA, whereas loin fat depth, tissue depth, rib fat depth, and shoulder fat depth did not differ (p>0.05) between the genotypes. Further, the mean shear value of cooked chops of F1-RCA lambs was higher (p<0.001) than AW lambs (Table-3). No differences (p>0.05) were observed among genotypes in terms of cooking loss and water holding capacity. The redness and pH values differed (p<0.05) between the genotypes, while yellowness and lightness did not (p>0.05).

## Discussion

The F1-RCA lambs grew faster than AW lambs during the fattening trial. However, these results disagree with other researchers [5,6,15-17] who reported no effect of the presence of the CLPG mutation on live weight during the post-weaning period. The highly significant effect of CLPG mutation on AW post-weaning growth rate in this study could be explained by the major effect of this gene on the hindquarters of the Rambouillet beyond the meat-type sheep as indicated in the previous studies [5,6,15,16,18]. In addition to the Rambouillet genes (50%) effect that may play a pivotal role in enhancing the growth of the results crossbred. These researchers obtained a non-significant effect of CLPG on growth rate, using thin fat tail meat-type sheep breeds (Dorset, Texel, and Suffolk) and their crosses with CLPG carriers. With the AW sheep, a fat tail dual purpose sheep breeds with considerable milk production [19], breed differences could account for the results obtained in our study. FCR was less for F1-RCA than AW lambs as indicated by Jackson *et al*. [5], where CLPG lambs had less FCR compared to normal half-sibling. The superior feed efficiency in the CLPG lambs may be explained by those CLPG lambs utilizing energy for protein rather than fat accretion [5]. A substantial effect (p<0.001) of CLPG mutation on carcass weights (hot and cold) was observed in Dorset rams heterozygous for the CLPG mutation as compared to normal lambs produced from Dorset or Texel rams, which is in agreement with Abdulkhaliq *et al*. [18]. Jackson *et al*. [10] reported that Rambouillet lambs expressing CLPG mutation had heavier hot and cold carcass weights than their normal half-siblings. The weight of shoulders, legs, racks, and loins was significantly higher in the F1-RCA than AW, similar to others [17,18,20,21]. The observed significant difference in the fat tail weight, higher in the AW (Table-2), is an expected result since the Rambouillet sheep breed is thin-tailed and the F1-RCA contains 50% of the Rambouillet genes. Unexpectedly, dressing percentage was not different between F1-RCA and AW lambs (p>0.05). Previous reports indicated greater dressing percentages for CLPG carcasses [6,10], as influenced by the amount of muscle, fat, gut fill, and wool on the lamb. Koohmaraie *et al*. [6] found higher dressing percentages for CLPG lambs influenced by the decrease in some internal organs weight. In our experiment, increased weight of non-carcass components in F1-RCA rather than dressing percent was observed in lambs slaughtered at 52 kg. The longissimus muscle weight and leg muscle were heavier (p<0.05) by 119% and 68% F1-RCA compared to the AW, respectively. Previously published results [6,7,10,21-24] indicated that individual muscle weights in the hind limbs and loin were heavier in lambs carrying the CLPG mutation compared to non-carrier controls. Differences in muscle weights in the leg and loin cuts of CLPG lambs may result from an increase in magnitude of expression of the CLPG mutation [10]. Koohmaraie *et al*. [6] linked the large muscle mass of the CLPG to the observed high RNA contents, increased satellite cell proliferation, and a capacity to synthesize protein combined with a reduction in protein degradation in the CLPG muscles (longissimus and Semitendinosus). Carpenter *et al*. [22] concluded that the large increase in muscle mass (semitendinosus, longissimus, and gluteus medius) in CLPG lambs was strongly associated with changes in the fast twitch glycolytic fibers, the only fiber type that increased in cell size as a percentage of the total fiber including slow-twitch oxidative (SO) and fast-twitch oxidative glycolytic fibers. There was no difference in the amount of intermuscular fat in leg between the genotypes (Table-3). These results did not agree with Carpenter *et al*. [22], who reported lower fat content in semitendinosus, longissimus, and gluteus medius muscles in the CLPG than in non-CLPG lambs, possibly the result of less intramuscular fat. The current results showed that the F1-RCA had more total bones. While bone-to-lean ratio was higher in pure AW, this finding concurs somewhat with the study by Jackson *et al*. [10]. Eye muscle area was 83% larger, 32% wider, and 61% greater in F1-RCA carcasses compared to the AW, in agreement with some previous results [6,7,15]. All linear fat measurements except shoulder fat thickness (S2) were significantly lower in F1-RCA when compared to AW, which agrees with findings reporting less fat thickness, fat depth, body wall thickness, and fat depth over loin in CLPG than normal lambs [6,7,15,18,20,21,25].

Further, the tenderness of the longissimus muscle was lower in the F1-RCA compared with the AW (19.0 vs. 7.2 kg). The higher shear force measurements in the muscles of the CLPG are consistent with the findings of previous reports [6,17,18,20,21,25]. Koohmaraie *et al*. [6] suggested that the reduction rate of protein degradation and the higher capacity for protein synthesis are consequences of the CLPG condition. These properties are also associated with lower meat tenderness resulting from reduced rate and extent of postmortem meat proteolysis. On the other hand, Abdulkhaliq *et al*. [17] suggested that cookery method may play a major role in the gene effect on meat tenderness. The increased toughness of CLPG meat has been attributed to higher calpastatin activity, which results in decreased protein degradation [6,26]. In this study, the cooking loss was not significantly different between the F1-RCA and AW meat. Duckett *et al*. [27] and Everts *et al*. [25] found no difference in cooking loss between CLPG lambs and normal lambs. No significant difference in water holding capacity was found between F1-RCA and AW. This is not in agreement with Abdulkhaliq *et al*. [17], who suggested that the moisture content of uncooked loin muscle was higher for CLPG lambs. There was a significant difference between F1-RCA and AW in the meat pH (p<0.05), as shown by Goodson *et al*. [21], where CLPG muscles had lower pH than normal muscles at various time points. Everts *et al*. [25] also found that pH values for CLPG longissimus muscles were lower than normal longissimus muscles. Warner *et al*. [28] inferred that an increase in the proportion of the glycolytic fibers in muscles of “CLPG” reduces oxidative metabolism and thus leads to pH variations.

Meat redness, but not color coordinates (L* and b*) differed between the F1-RCA and AW meat, with F1-RCA less red than AW. Abdulkhaliq *et al*. [17] found that the CLPG longissimus muscles were more yellow in color (p<0.01) and slightly less red than normal lambs. Kerth *et al*. [26] also found that supraspinatus, longissimus, and semitendinosus muscle color was lighter for CLPG carcasses when compared with normal carcasses. In another hand, the loins of the CLPG lamb have stability of color, while exhibiting lower tenderness compared to loins from non-CLPG phenotype lambs [29].

## Conclusion

The growth performance of AW sheep has been improved in the first generation of F1-RCA sheep carrying the CLPG mutation. The CLPG in 50% Rambouillet with 50% AW genes can, therefore, be used in structured mating systems to make dramatic improvements in growth rate, feed efficiency, ADG, and carcass composition of AW sheep. A backcross generation using the F1-RCA rams with pure AW ewes should be conducted to retrieve the AW phenotype and to reduce the Rambouillet percentage in the newly formed crossbred AW-Rambouillet population. The confounding effects of heterosis, Rambouillet, and CLPG of this first cross should be investigated in a new backcross generation.

## Authors’ Contributions

KIZJ: Designed the experiment, supervised the field work, data collection, statistical analysis, interpretation of results, and drafting the manuscript, AHA: Fieldwork and data collection, and PYA: Revised the English writing of the manuscript and interpretation the obtained results. All authors read and approved the final manuscript.
